# Cortical Manifestations in Capsular Warning Syndrome: Expanding the Clinical Spectrum With a Case of Subcortical Aphasia

**DOI:** 10.7759/cureus.99230

**Published:** 2025-12-14

**Authors:** Karina Angela G Lipardo, Jo Ann R Soliven

**Affiliations:** 1 Department of Neurology, The Medical City, Pasig, PHL

**Keywords:** capsular warning syndrome, cortical hypoperfusion, internal capsule infarction, ischemic stroke, rtpa, subcortical aphasia, thrombolysis

## Abstract

Capsular warning syndrome (CWS) is a form of transient ischemic attack characterized by repetitive episodes of sensorimotor deficits within 24 hours, typically involving subcortical ischemia of the internal capsule without cortical features. We report a case of a 75-year-old right-handed female who presented with fluctuating neurological deficits, including Broca’s aphasia, right-sided hemiplegia, right central facial palsy, and right hemianesthesia. She experienced seven episodes of waxing and waning symptoms lasting from five minutes to an hour, with National Institutes of Health Stroke Scale (NIHSS) scores ranging from 0 to 17. Computed tomography (CT) was unremarkable, and she received intravenous recombinant tissue plasminogen activator (rTPA) four hours after the initial onset of symptoms, eventually resulting in the resolution of aphasia but with residual hemiplegia. Magnetic resonance imaging (MRI) performed 24 hours later revealed acute infarction of the left internal capsule, caudate nucleus, and lentiform nucleus. This case underscores the importance of recognizing atypical presentations of capsular warning syndrome in the emergency room, which may indicate reversible dysfunction that may respond to timely reperfusion therapy.

## Introduction

Capsular warning syndrome (CWS), first described in 1993, is a form of transient ischemic attack characterized by three or more transient episodes of motor or sensorimotor dysfunction within 24 hours, with complete resolution between events [1]. Later studies, however, have extended this time window up to 72 hours [2]. This occurs due to ischemia of the internal capsule or other subcortical areas; hence, CWS classically does not present with cortical signs such as aphasia, neglect, or dyspraxia [1]. However, evidence shows that subcortical lesions may produce cortical deficits, such as aphasia, due to disruption of subcortical-cortical circuits or temporary cortical hypoperfusion [3,4]. In this case report, we describe a case of CWS with expressive aphasia that improved with thrombolytic therapy, highlighting the need for early recognition of atypical presentations of CWS, which may indicate reversible dysfunction.

## Case presentation

A 75-year-old right-handed woman with hypertension and dyslipidemia, who was fully independent in all activities of daily living, experienced sudden onset of right-sided weakness, facial asymmetry, and expressive aphasia at 4:50 PM. She arrived at the emergency department 17 minutes later with complete resolution of symptoms and a National Institutes of Health Stroke Scale (NIHSS) Score of 0. Five minutes after her arrival, however, the patient was noted to have a recurrence of the deficits (Table 1). She now had an NIHSS score of 17, with her deficits including right-sided hemiplegia (motor strength of right upper extremity 0/5, and right lower extremity 1/5), right central facial palsy, right hemianesthesia, and expressive aphasia. The patient had a blood pressure of 250/220 mmHg and was given nicardipine 2 mg intravenously, resulting in an improvement to 173/76 mmHg. A computed tomography (CT) scan with angiography was performed, and the initial results were negative for infarct, hemorrhage, and thrombus formation. Thrombolysis was initially considered as treatment, but was eventually deferred since the symptoms had resolved 23 minutes later, and the patient’s NIHSS score had returned to 0.

**Table 1:** Temporal Profile of Recurrent Neurological Deficits *The NIHSS of the patient during the first episode was not recorded since the deficits had already resolved upon arrival of the patient at the emergency room. Abbreviations: NIHSS = National Institutes of Health Stroke Scale

Episode	Onset	Time Resolved	Interval	Duration	NIHSS
1	4:50 PM	5:07 PM	—	17 min	?*
2	5:12 PM	5:35 PM	5 min	23 min	17
3	8:53 PM	9:35 PM	198 min	42 min	17
4	9:45 PM	9:50 PM	10 min	5 min	15
5	11:14 PM	11:25 PM	84 min	11 min	15
6	12:55 AM	1:55 AM	90 min	60 min	15
7	2:15 AM	—	20 min	—	11

However, four hours and three minutes after the initial ictus, the patient had a recurrence of the same deficits for a third time, with an NIHSS score of 17. Though thrombolysis was initially deferred earlier, the recurrence of symptoms and a rising NIHSS score suggested possible progression to a fulminant stroke with lasting deficits. Therefore, recombinant tissue plasminogen activator (rTPA) was administered at four hours and five minutes after initial symptom onset at a dose of 0.9 mg/kg. Post-thrombolysis, the patient was admitted to the acute stroke unit, where she continued to experience waxing and waning neurological deficits over the next 10 hours (Table 1). Her blood pressure ranged from 120-190/60-70 mmHg, without any episodes of hypotension. She experienced seven discrete events, with documented durations ranging from five minutes to 60 minutes, except for the seventh episode, which had no resolution or measurable duration as her deficits persisted thereafter. Symptom-free intervals varied widely, from five minutes to 198 minutes.

**Figure 1:**
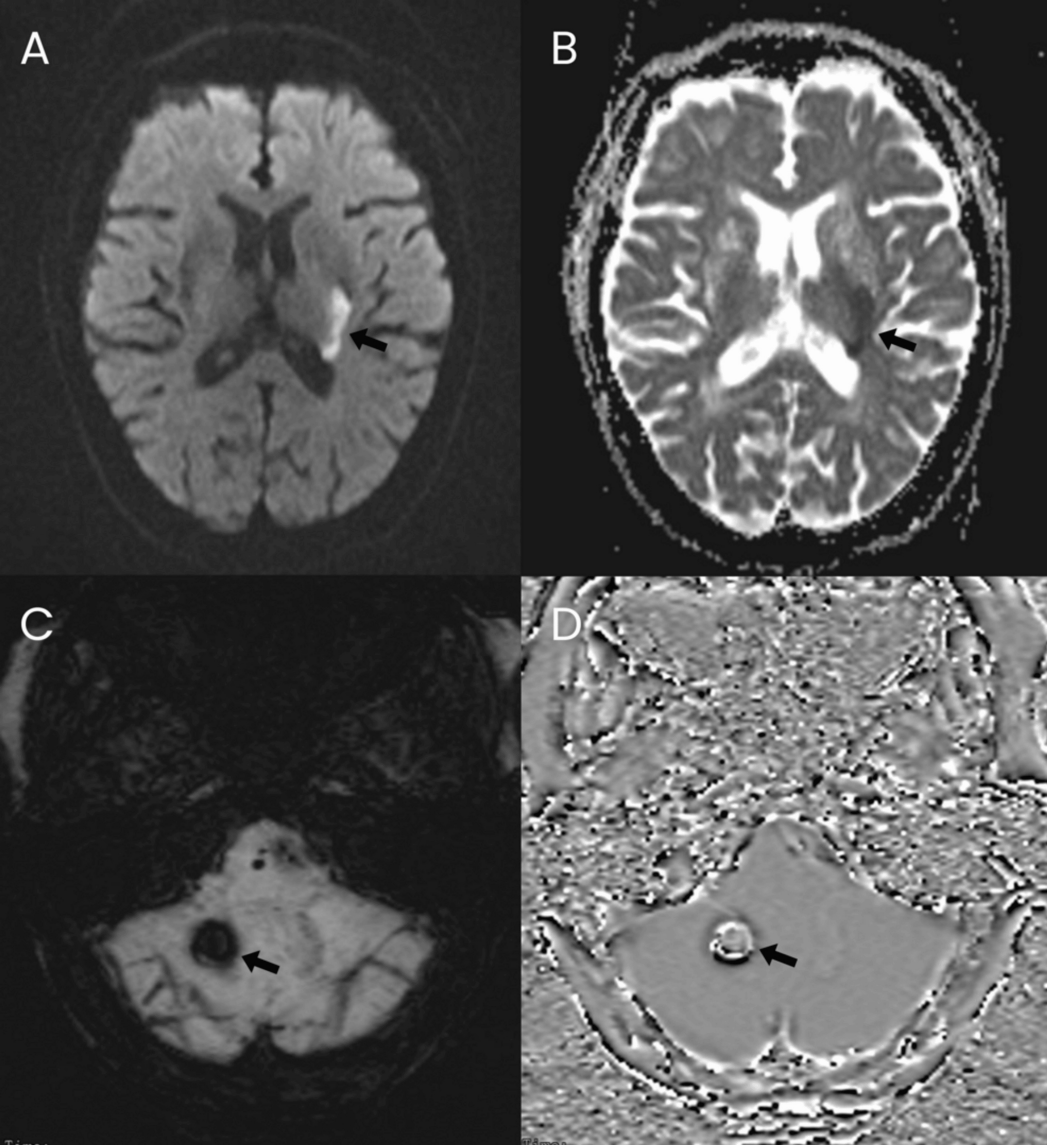
MRI findings. Axial DWI (A) and ADC (B) show restricted diffusion involving the left caudate body, posterior limb of the internal capsule, and lentiform nucleus (arrows), consistent with acute infarction. SWI (C) and filtered-phase imaging (D) demonstrate a small acute hemorrhage in the inferior right cerebellar hemisphere (arrows). Abbreviations: DWI, diffusion-weighted imaging; ADC, apparent diffusion coefficient; SWI, susceptibility-weighted imaging.

**Figure 2:**
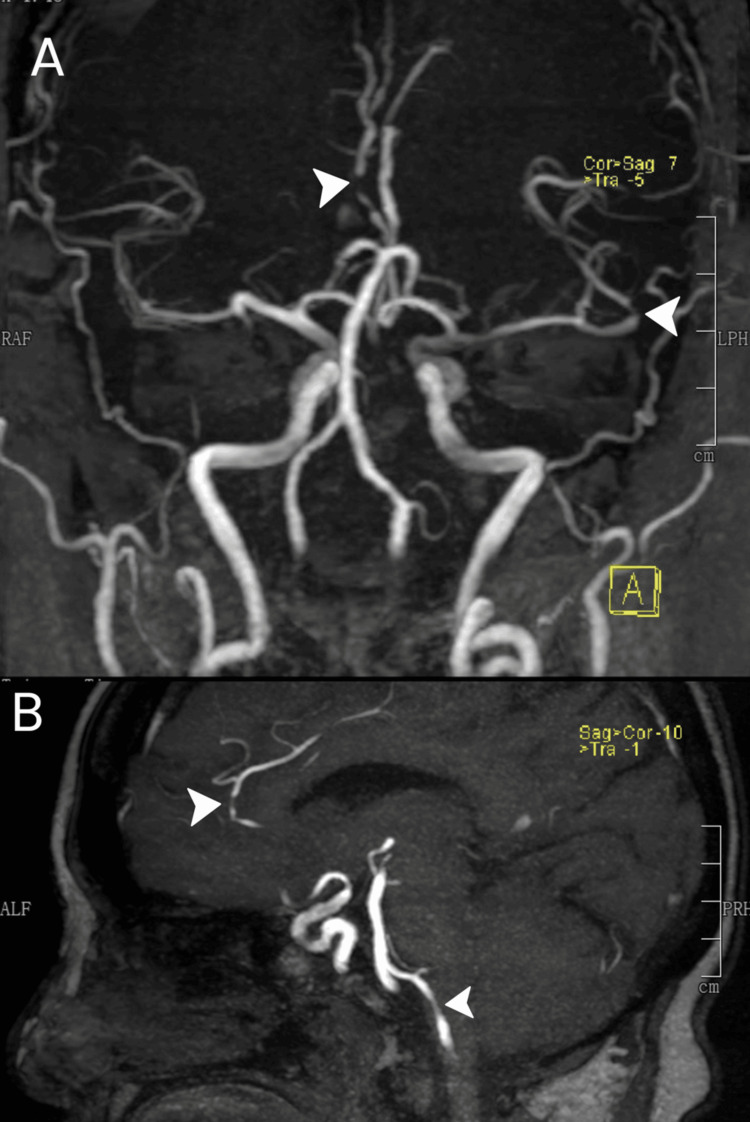
MRA findings. Time-of-flight MRA (A) in a coronal MIP shows mild short-segment stenosis of the distal A2 segment of the right anterior cerebral artery (superior arrowhead) and narrowing of the proximal inferior division M2 segment (inferior arrowhead). A sagittal oblique MIP (C) demonstrates mild narrowing of the left V4 vertebral artery (inferior arrowhead) and again shows the distal right A2 stenosis (superior arrowhead). MRA, magnetic resonance angiography; MIP, maximum-intensity projection.

After 24 hours, the patient had complete resolution of expressive aphasia. However, she still had other sensorimotor deficits, with an NIHSS score of 11, characterized by dysarthria, right central facial palsy, right hemiplegia (motor strength of right upper extremity 0/5, and right lower extremity 1/5), and right hemianesthesia. Repeat neuroimaging with a plain cranial MRI with angiography (MRA) was done. This showed an acute infarction of the posterior limb of the left internal capsule, left caudate nucleus, and left lentiform nucleus (Figure 1A, 1B). There was also an acute hemorrhage on the right inferior cerebellar hemisphere (Figure 1C, 1D). MRA revealed multiple mild short-segment stenoses in several vessels: the distal A2 segment of the right anterior cerebral artery, proximal inferior division M2 segment, posterior opercular branch of the left middle cerebral artery, and V4 segment of the left vertebral artery (Figure 2). While admitted, she started physical and occupational therapy. Antiplatelet medications were initially withheld due to intracerebral hemorrhage, but dual antiplatelet therapy with aspirin and clopidogrel was eventually started in the outpatient setting. The patient was discharged after one week with a persistent NIHSS score of 11, reflecting the persistent sensorimotor deficits as previously described, but without recurrence of expressive aphasia.

## Discussion

This case broadens the recognized clinical spectrum of CWS by demonstrating that cortical features such as expressive aphasia can occur despite a purely subcortical lesion. Understanding how a subcortical infarct can produce a cortical deficit requires examining the potential mechanisms linking deeper structures to cortical language networks.

Aphasia, or a language deficit, is generally considered a cortical symptom, but various studies have reported aphasia following subcortical lesions in the thalamus, internal capsule, or striatum, suggesting that these regions play a crucial role in language processing [3,4]. Two major mechanisms have been proposed. First, injury to subcortical structures may directly disrupt subcortical-cortical language circuits. Second, transient cortical hypoperfusion may impair function in language-dominant cortical regions despite the absence of cortical infarction [4,5]. Cortical hypoperfusion is often not visible on CT or MRI and requires perfusion-weighted imaging (PWI). In acute stroke, there may be a mismatch between the infarcted area on MRI and the area of hypoperfusion on PWI. Cortical hypotension may progress to cortical infarction, but many cases are transient, and aphasia symptoms are reversible [4]. In our case of capsular warning syndrome, there was a complete reversal of the aphasia, indicating cortical reperfusion after rTPA.

Beyond mechanisms of aphasia, the broader pathophysiology of CWS remains incompletely understood. CWS has long been thought to reflect hemodynamic instability within small perforating arteries, leading to fluctuating ischemia in subcortical territories. Hypertension and dyslipidemia, both present in our patient, are the most common risk factors and can cause microvascular changes such as lipohyalinosis or microatheroma formation [1,6,7]. Recently, however, large-artery atherosclerosis, such as middle cerebral artery stenosis, has also been hypothesized as a possible cause [8]. In our case, the presence of multiple mild stenoses on MRA raises the possibility of intracranial atherosclerotic stenosis (ICAS) as an underlying mechanism. ICAS is more prevalent among Asian populations and represents one of the leading etiologies of stroke in this demographic [9]. Its hemodynamic consequences - including impaired distal perfusion and susceptibility to fluctuating ischemia [10] - align well with the pathophysiology of capsular warning syndrome. Considering ICAS has important implications for secondary stroke prevention, which extends beyond standard antiplatelet therapy to include aggressive vascular risk-factor modification, high-intensity statin therapy, and short-term dual antiplatelet therapy [10,11]. Recognizing this mechanism may therefore guide more tailored long-term management strategies for preventing stroke recurrence.

The exact mechanism of the fluctuating symptoms in CWS remains unclear, but it is thought that generalized hemodynamic instability ultimately leads to intermittent ischemia [1,7,12].

Management of CWS, particularly the role of thrombolysis, remains a topic of debate. Initial treatment of CWS in the literature includes intravenous thrombolysis, anticoagulant therapy, antiplatelet drugs, and vasopressors; each targeting different proposed mechanisms of endothelial dysfunction or hypoperfusion [6,7]. While several case reports, including ours, document improvement after rTPA, larger retrospective studies have not shown a statistically significant difference between thrombolyzed and non-thrombolyzed patients [7]. It is important to note that most available studies on the treatment of CWS are retrospective, and randomized clinical trials have yet to be done. The recommended timing of rTPA in CWS is also not clearly stated in previous studies, but according to ESO and AHA/ASA guidelines, rTPA should be given 3-4.5 hours from stroke symptom onset or last known well [13,14]. In cases of CWS, the true ictus is still considered as the time of onset of the first episode of deficits. It is unclear whether the ictus can be reset to the time of the most recent episode. However, in a previous case report, rTPA was given to a patient with CWS 13 hours after the first episode and 80 minutes after the most recent episode, with notable symptom improvement [12]. It is up to the clinician’s assessment to give rTPA if these deficits are deemed disabling despite their fluctuations, such as aphasia or dense hemiplegia. MRI should ideally be done for CWS patients to be able to visualize if the CWS has already progressed into an infarct. However, in resource-limited areas where only a CT scan is available, as long as hemorrhage has been ruled out and there are no other contraindications, rTPA may be given. An additional factor that could influence treatment decisions in this case is premorbid frailty, given the older age of the patient. Frailty has emerged as a significant predictor of outcomes following intravenous thrombolysis, with evidence suggesting that frail older adults experience higher rates of complications and reduced functional recovery [15]. Although the patient was 75 years old, she was functionally independent prior to her stroke, and there were no indications of significant premorbid frailty. This supported the decision to administer thrombolysis despite fluctuating symptoms.

To our knowledge, no previous literature has reported CWS presenting with cortical manifestations. Although the term “capsular warning syndrome” traditionally implies a capsular lesion, other warning syndromes, such as pontine warning syndrome, have been documented, demonstrating that the phenomenon is not confined to a single anatomical location [16]. Thus, clinicians should identify these atypical manifestations as warning syndromes, provided they meet the other criteria: three or more repetitive episodes of neurological deficits within 24-72 hours. Importantly, if these patients present at the emergency room within the therapeutic window, early consideration of thrombolysis may prevent irreversible neurological deficits despite fluctuations in symptom severity.

## Conclusions

This case demonstrates that CWS can present with cortical deficits such as expressive aphasia, likely due to transient cortical hypoperfusion. The full reversal of aphasia after rTPA highlights the potential for recovery when reperfusion therapy is given promptly. Clinicians should recognize that atypical features do not exclude CWS as a diagnosis, and hence should consider thrombolysis for fluctuating but disabling symptoms to prevent permanent neurological deficits.
